# Editorial: Thyroid nodules and tumors in childhood

**DOI:** 10.3389/fendo.2023.1303749

**Published:** 2023-10-19

**Authors:** Luisa de Sanctis, Malgorzata Wasniewska, Maria Cristina Vigone

**Affiliations:** ^1^ Department of Public Health and Pediatric Sciences, University of Torino, Torino, Italy; ^2^ Department of Human Pathology in Adulthood and Childhood, University of Messina, Messina, Italy; ^3^ Department of Pediatrics, IRCCS San Raffaele Scientific Institute, Milan, Italy

**Keywords:** thyroid cancer, thyroid nodules, thyroiditis, del22q11 syndrome, phosphatase and tensin homolog deleted on chromosome 10 (PTEN), EU-TIRADS, FNAB, ACR-TIRADS

Nodular thyroid disease in pediatric age displays a lower prevalence than in adulthood, even if to date no literature data have been produced on the global incidence and prevalence of this condition in childhood. Although most thyroid nodules in childhood are benign, they carry a risk of malignancy up to five times greater than that in adults (1, 2). Most pediatric patients with differentiated thyroid cancer (DTC) present with an asymptomatic thyroid nodule found occasionally; not fully investigated are the association with autoimmune thyroiditis and the correlation with US changes over time (3). To standardize medical decisions and avoid unnecessary fine needle aspiration biopsy (FNAB), the European Thyroid Association and the American Radiology College have established guidelines for Thyroid Imaging, Reporting and Data System (EU-TIRADS and ACR-TIRADS). Based on cytological finding on FNAB, several classifications have been produced, of which the Bethesda System for Reporting Thyroid Cytopathology (BSRTC) and the British Thyroid Association (BTA) are the most widely used. However, increasing evidence, using BSRTC and BTA (3), or other National cytological classifications (4), indicates the need for pediatric-specific data for the optimal management of thyroid nodules in children, which may differ from that of adult nodules with equivalent cytology. Furthermore, the broadening of knowledge of the oncogenic landscape supports the incorporation of oncogene testing to rule-in malignancy of nodules with indeterminate cytology. Finally, in patients with syndromes with genetic predisposition, a surveillance approach is necessary to promptly recognize a DTC occurrence.

Aim of this Research Topic was to collect evidence on epidemiology, diagnostic tools and pathways, as well as data on the management and long-term outcomes of “*Thyroid nodules and tumors in childhood”*, with a focus also on possible correlation with autoimmune disease and rare genetic syndromes with predisposition to thyroid malignancies. The present Research Topic includes 1 *Systematic Review*, 6 *Original articles* and 2 interesting *Case reports* on rare diseases with genetic predisposition to thyroid malignancies, thus covering most of the current research in the specific field of thyroid nodules and malignancy in the pediatric age.

To estimate the overall incidence and prevalence of DTCs in the pediatric age and seek for differences between sub-types, i.e. the more frequent papillary thyroid carcinoma (PTC) and the rare follicular thyroid carcinoma (FTC), Moleti et al. conducted a systematic review of articles produced from 2000 to 2021 and two separate meta-analysis. They provide valuable data on the current global epidemiology of pediatric and adolescent DTC and indicate the need for a prospective international registry on pediatric DTC, based on standardized data collection, to implement relevant information on the clinical behavior of these rare diseases.

In an original article, Tuli et al. focused their research on the effectiveness of the EU-TIRADS and ACR-TIRADS scoring systems in risk stratification in a large pediatric population of 200 patients with thyroid nodules referred to a single Center over a 20-year period, adding new data on the performance and the limitations of these two important diagnostic tools used routinely in the clinical practice, also compared with the FNAB results.

Januś et al. provided two interesting papers from their Center; in the first, through a prospective follow-up study, they report their experience on the US evolution of the thyroid gland with autoimmune thyroiditis (AIT) before the development of PTC, in a population of 180 children referred to their Outpatient Endocrine Department for suspected thyroid disorder, confirming the fundamental role of thyroid US in the routine follow-up of patients with AIT, not only for the early detection of clinically-silent thyroid malignancies, but also for the possible influence on oncological therapy. In the second paper, through a retrospective analysis, they indicate their 22-year experience on the natural course and US, laboratory, and histopathological features on a cohort of 90 pediatric patients with PTC diagnosed through the EU-TIRADS scoring system, with and without AIT.

A similar issue was investigated by Jie et al., who compared the US, clinical, and pathological features of 52 children and adolescents with PTC, with and without Hashimoto’s thyroiditis (HT), indicating HT as a possible independent risk factor in children and adolescents with PTC, which showed more aggressive features.

Sarli et al., through a multicenter survey, underline the possible risk of thyroid neoplasms in patients with rare disorders of the neck region, i.e. the 22q11.2 deletion and DiGeorge-like syndromes, conditions rarely associated with malignancies, for which no aggregate data exist, thus emphasizing the need for prolonged clinical and US follow up for these conditions.

In their rigorous manuscript van de Berg et al., within a retrospective analysis of nationwide population-based data, summarized the long-term oncological outcomes of the two subtypes of the DTC, the PTC and FTC, in the Dutch population aged <18 years, diagnosed between 2000 and 2016, with important advances on the knowledge of the trend over time of these 2 forms of DTC, of any risk factors for recurrence and of survival rate.

The Research Topic also contains 2 very peculiar *Case Reports* of great clinical value for the diagnostic pathway, the genotype-phenotype correlation and impact on family counseling.

The first, produced by Vincenzi et al., reports the occurrence of a multinodular goiter in a patient with Bannayan-Riley-Ruvalcaba syndrome associated with a PTEN variant and congenital hypothyroidism due to homozygous alterations of the TPO gene, arguing a possible synergic role of TPO and PTEN mutations, a gene known to predispose to thyroid cancer (5).

The second, reported by Stambouli et al., describes the appearance of a rare embryonal rhabdomyosarcoma in a patient found to harbor a germline DICER1 mutation, highlighting the need to look for DICER1 syndrome in the presence of rare and unusual tumors during childhood, in the presence of a family history of thyroid diseases in childhood or early adulthood.

In conclusion, this Research Topic contains original contributions that can enrich scientific knowledge and help improve daily clinical practice and follow-up of patients with nodular and tumor disorders of thyroid in childhood; it can also represent a food for thought and inspiration for new research in this area.

## Author contributions

LS: Writing – original draft, Writing – review & editing. MW: Writing – review & editing. MV: Writing – review & editing.
